# Supplementary material for: Cross-neutralizing antibodies bind a SARS-CoV-2 cryptic site and resist circulating variants
Source: Nat Commun. 2021 Sep 27;12:5652. doi: 10.1038/s41467-021-25997-3 (PMC8476643; doi:10.1038/s41467-021-25997-3)
Supplement: Supplementary file 1 — Supplementary Information [file 41467_2021_25997_MOESM1_ESM.pdf]

Supplementary Information

Cross-neutralizing antibodies bind a SARS-CoV-2 cryptic site and resist circulating variants

Tingting Li[1,2,†], Wenhui Xue[1,2,†], Qingbing Zheng[1,2,†], Shuo Song[3,4,†], Chuanlai Yang[1,2,†], Hualong Xiong[1,2,†], Sibo Zhang[1,2], Minqing Hong[1,2], Yali Zhang[1,2], Hai Yu[1,2], Yuyun Zhang[1,2], Hui Sun[1,2], Yang Huang[1,2], Tingting Deng[1,2], Xin Chi[1,2], Jinjin Li[1,2], Shaojuan Wang[1,2], Lizhi Zhou[1,2], Tingting Chen[1,2], Yingbin Wang[1,2], Tong Cheng[1,2], Tianying Zhang[1,2], Quan Yuan[1,2], Qinjian Zhao[1,2], Jun Zhang[1,2], Jason S. McLellan[5], Z. Hong Zhou[6,7,*], Zheng Zhang[3,4,*], Shaowei Li[1,2,*], Ying Gu[1,2,*], Ningshao Xia[1,2,8,*]

Correspondence to: nsxia@xmu.edu.cn (N.X.) or guying@xmu.edu.cn (Y.G.) shaowei@xmu.edu.cn (S.L.) or zhangzheng1975@aliyun.com (Z.Z) or hong.zhou@ucla.edu (Z.H.Z)

This PDF file includes:

Figs. S1 to S5
Tables S1 to S6
References (1-7)

Supplementary Figures

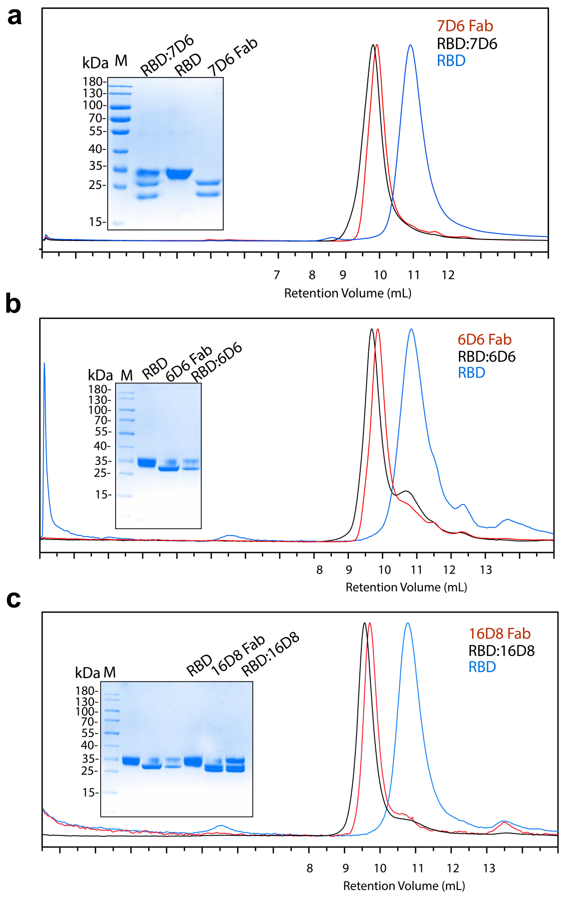

Fig. S1 HPLC profiles and SDS-PAGE analysis of RBD:7D6, RBD:6D6 and RBD:16D8 complexes. The experiments in **a, b** and **c** were performed twice with similar results. Source data are provided as a Source Data file.

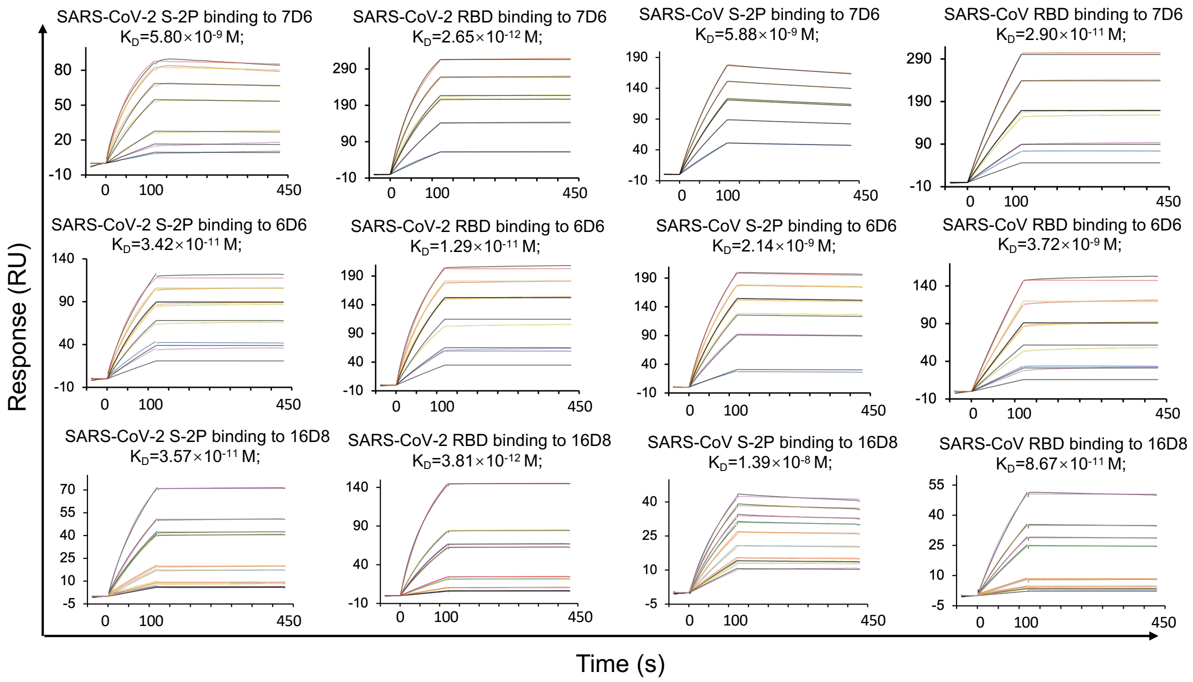

Fig. S2 Binding curves of SARS-CoV-2 and SARS-CoV RBD and S-2P proteins with 7D6, 6D6 or 16D8 antibodies in SPR. The kinetic constants are reported in Table S2. Color curves are the experimental traces obtained from surface plasmon resonance (SPR) experiments, and curves indicate the best global fit for the data used to calculate the K_D values using 1:1 binding model.

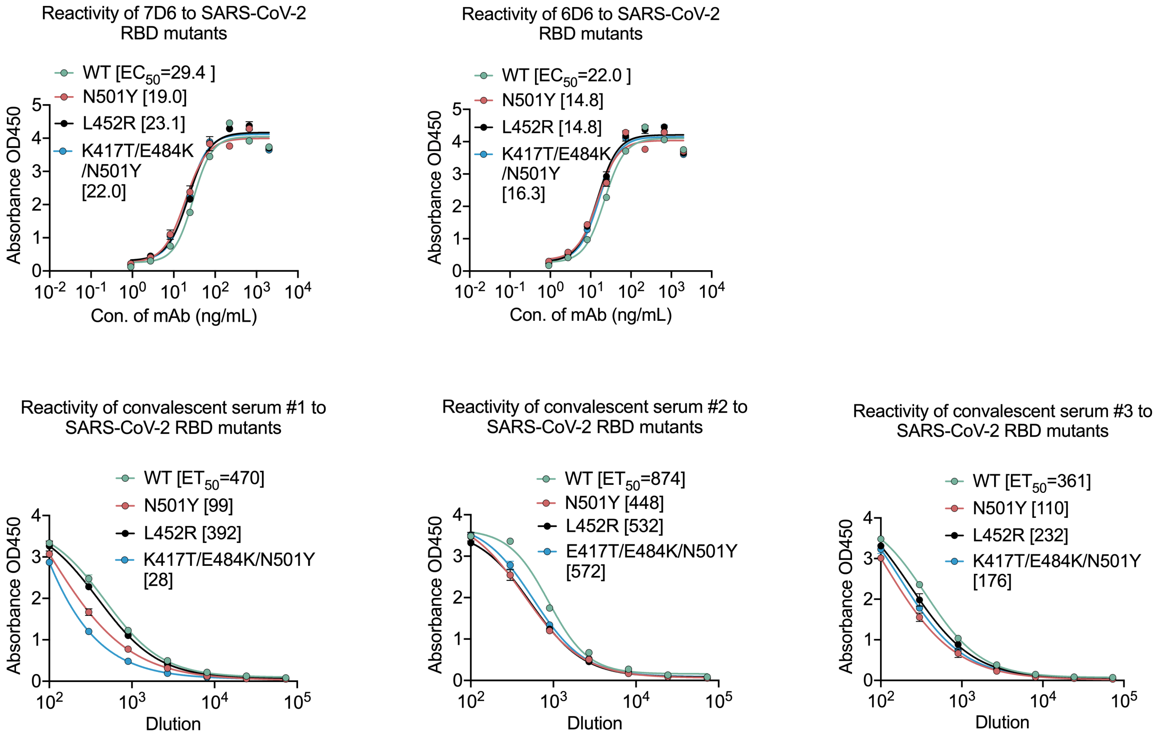

Fig. S3 Binding reactivities of 7D6, 6D6 and COVID-19 convalescent sera against the RBD mutants. COVID-19 convalescent sera exhibited lower reactivities to RBD mutants to various extents, the reactivities of serum #1 and #2 to mutants decreased by 2-20 folds compared to wild-type (WT) RBD. Data are presented as mean values +/- SEM.

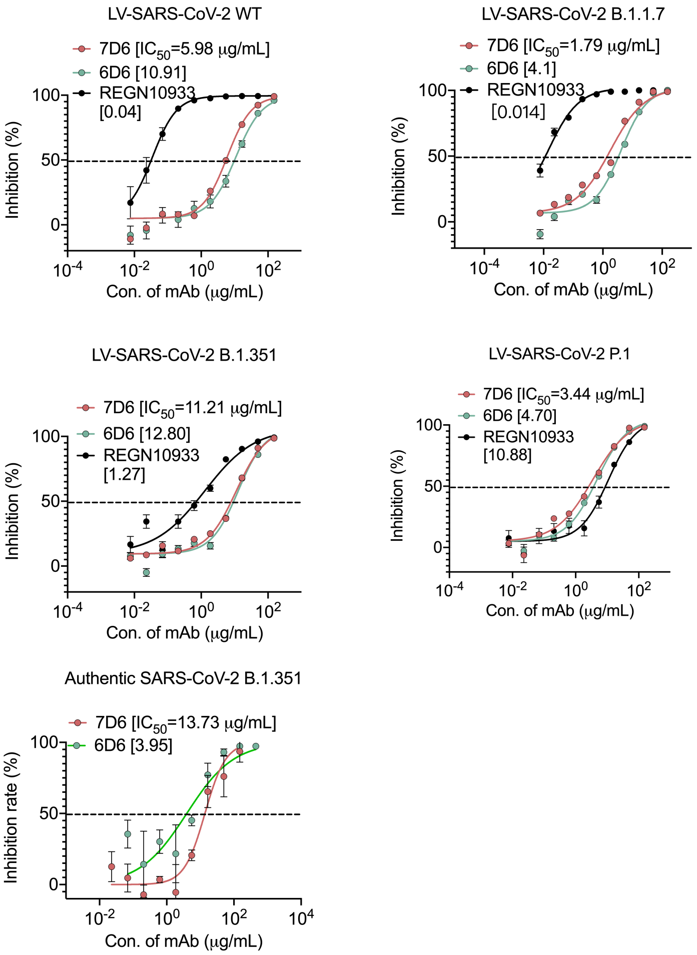

Fig. S4 Neutralization activities of 7D6, 6D6 against pseudotyped LVs and authentic virus of the major SARS-CoV-2 variant(s). Data are presented as mean values +/- SEM.

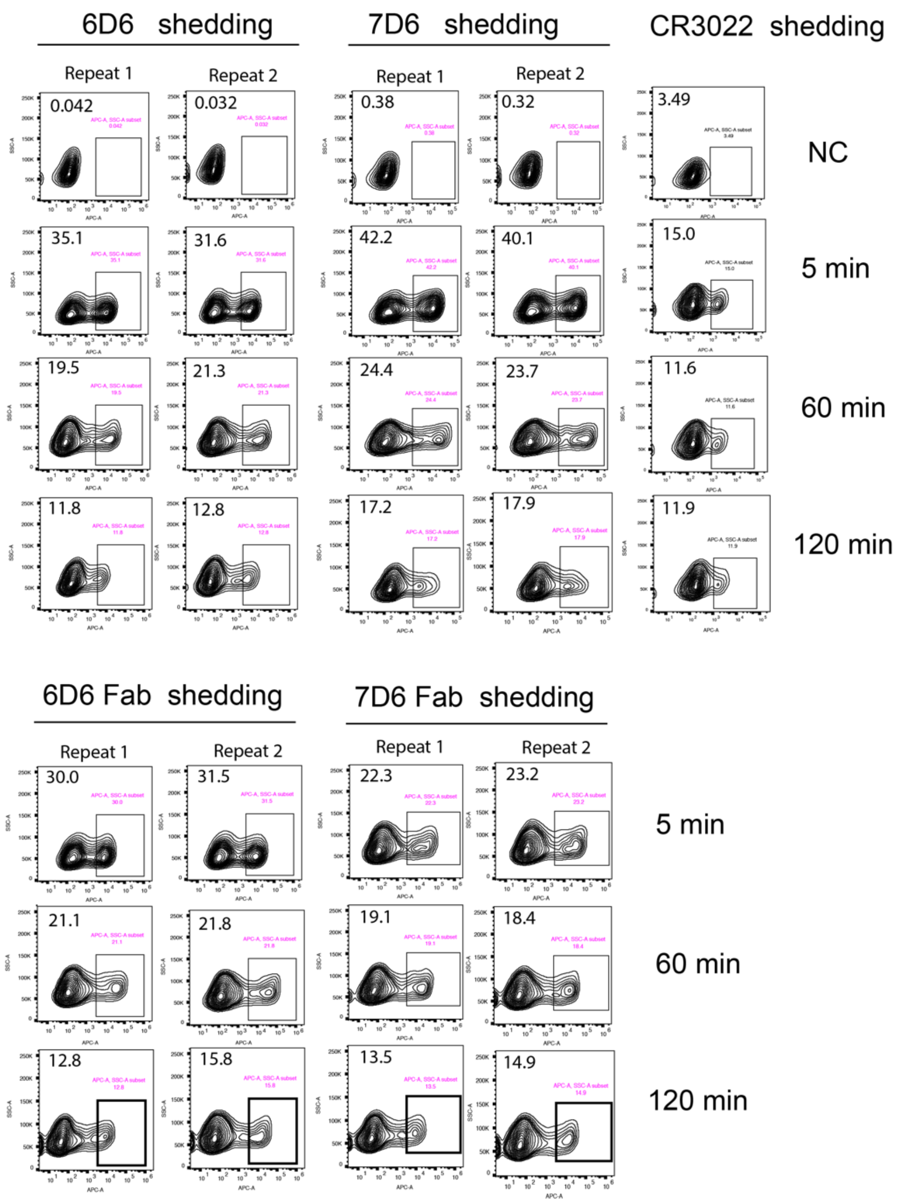

Fig. S5 The shedding property of 7D6 and 6D6. Shedding of S1 was measured by flow cytometry with 293T cell-surface expressed wildtype SARS-CoV-2 spike protein (S-WT). The percentage of cells at each collected time point was determined by the number of positive cells in the selected gates. CR3022 was used as the control.

Supplementary Table

Table S1 Binding activities and neutralizing titers of SARS-CoV-2 antibodies.

	EC50 (ng/mL)					IC50 (μg/mL)		
mAb	**SARS-CoV-2 S-2P binding**	**SARS-CoV S-2P binding**	SARS-CoV-2 S1 binding	RBD binding	S2 binding	**SARS-CoV-2 LV**	**SARS-CoV LV**	SARS-CoV-2 VSV
7D6	**11.28**	**12.86**	12.22	7.42	>10,000	**2.56**	**10.11**	0.04
5A12	**23.81**	**11.13**	>10,000	>10,000	15.00	**>150**	**>150**	0.76
13F1	**21.05**	**27.00**	>10,000	>10,000	87.31	**>150**	**ND**	>150
12F7	**137.00**	**343.00**	>10,000	>10,000	81.39	**>150**	**ND**	>150
12F9	**30.68**	**286.8**	>10,000	>10,000	40.36	**>150**	**ND**	>150
6D6	**21.78**	**120.00**	39.32	68.02	>10,000	**8.91**	**1.67**	0.21
16D8	**19.96**	**44.06**	44.57	111.7	>10,000	**3.52**	**1.2**	0.26
19F10	**24.62**	**39.59**	44.25	33.97	>10,000	**1.06**	**2.67**	1.08
17F10	**35.20**	**26.29**	57.53	43.5	>10,000	**1.58**	**0.44**	ND
4A5	**42.40**	**87.80**	46.16	90.3	>10,000	**0.62**	**57.56**	ND
10D1	**29.35**	**25.84**	49.66	88.77	>10,000	**2.94**	**18.12**	ND
20H2	**45.57**	**32.97**	>10,000	>10,000	45.57	**>150**	**>150**	>150
8G5	**28.82**	**44.61**	46.16	84.25	>10,000	**>150**	**>150**	ND
5E7	**318.50**	**670.20**	581.80	217.60	>10,000	**>150**	**>150**	>150
1B11	**80.42**	**17.48**	72.93	>10,000	>10,000	**>150**	**>150**	>150

*ND denoted not detected.

Table S2 Kinetic constants for binding activities of antibodies to SARS-CoV-2 or SARS-CoV proteins.

mAb	Proteins	ka ($M^{-1}S^{-1}\times10^{4}$)	kd ($s^{-1}\times10^{-5}$)	K_D (nM)
7D6	SARS-CoV-2 S-2P	1.52	8.81	5.89
	SARS-CoV S-2P	4.78	24.10	5.04
	SARS-CoV-2 RBD	18.00	0.11	<0.01
	SARS-CoV RBD	5.85	0.17	0.03
6D6	SARS-CoV-2 S-2P	12.30	0.42	0.03
	SARS-CoV S-2P	3.06	5.56	2.14
	SARS-CoV-2 RBD	11.50	0.15	0.01
	SARS-CoV RBD	1.70	0.63	0.37
16D8	SARS-CoV-2 S-2P	3.55	0.18	0.04
	SARS-CoV S-2P	65.60	9.14	13.90
	SARS-CoV-2 RBD	3.93	0.02	<0.01
	SARS-CoV RBD	3.15	0.27	0.08

Table S3 Data collection and refinement statistics for 7D6:RBD and 6D6:RBD complexes.

	**7D6:RBD**	**6D6:RBD**
Data collection		
Cell parameters (Å, º)	a=37.4, b=88.0, c=102.7	a=58.2, b=85.3, c=160.7
	α=89.9, β=87.1, γ=89.9	α=90, β=90, γ=90
Space group	P1	$P2_12_12_1$
Resolution range [a] (Å)	29.32-1.40 (1.44-1.40)	30.08-1.91 (1.96-1.91)
Wavelength (Å)	0.97918	0.97918
Observed hkl (I>σ)	827,533	419,267
Unique hkl	244,264	62,975
Redundancy	3.4 (2.7)	6.7 (6.5)
Completeness (%)	94.7 (89.5)	99.8 (99.7)
Overall (I/σI)	8.8 (0.8)	13.7 (0.9)
R_{sym} [b] (%)	6.1 (124.7)	6.4 (216.7)
R_{pim} [c] (%)	3.9 (93.6)	2.7 (91.3)
CC1/2	0.996 (0.331)	0.999 (0.402)
Refinement		
Resolution range (Å)	21.68-1.40	28.67-1.91
Number of Reflections	244,139	62,867
R_{factor} [d]	17.2	18.9
R_{free} [e]	18.2	22.4
RMSD bond lengths (Å)	0.009	0.014
RMSD bond angles (°)	1.35	1.32
No. atoms	11,084	5,036
Protein	9,836	4,853
Glycan	84	14
Water	1,164	169
Wilson B-factor (Å^2)	22.7	42.9
Average B-factors (Å^2)	38.2	57.7
Protein	37.0	57.7
Glycan	104.3	113.4
Water	43.2	53.5
Ramachandran Plot		
Favored and allowed region (%)	97.0	97.9
Generously allowed regions (%)	2.6	1.9
Disallowed regions (%)	0.4	0.2

[a] Numbers in parentheses refer to the highest resolution shell.

[b] $R_{sym} = \Sigma h\ \Sigma i|I_l(h) - \langle I(h)\rangle| / \Sigma h\ \Sigma i\ I_l(h)$

[c] $R_{pim} = \Sigma h SQRT(1/(N-1))\ \Sigma i|I(_l h) - \langle I(h)\rangle| / \Sigma h\ \Sigma i\ I\ (_l h)$

[d] $R_{factor} = \Sigma hkl||Fobs| - k|Fcalc||/\Sigma hkl|Fobs|$.

[e] R_{free} is calculated using the same equation as that for R factor but 10.0% of reflections were chosen randomly and omitted from the refinement

Table S4 Conservation of 7D6 and 6D6 epitopes.

SARS-CoV-2 positions	Conservation	SARS-CoV and Other SARS-related	Conservation	Conservation in *Sarbecovirus*	Average conservation *	Average conservation of 7D6/6D6 site
R346	>99.8%	K/R	71.1%	85.5%	84.7%	89.0%
**Y351**[#]		Y	95.2%	97.5%		
**A352**		A	95.2%	97.5%		
**W353**		W	95.2%	97.5%		
**N354**		N	12.1%	56.0%		
**R355**		R	95.2%	97.5%		
K356		K	59.0%	79.4%		
**R357**		R/K	79.2%	89.5%		
T393		T	44.5%	72.2%		
**N394**		N	59.0%	79.4%		
**Y396**		Y	95.2%	97.5%		
P426		P	95.2%	97.5%		
R457		R	95.2%	97.5%		
S459		S	24.1%	62.0%		
N460		N	4.8%	52.3%		
**K462**		R/K	89.2%	94.5%		
P463		P	95.2%	97.5%		
**F464**		F	90.3%	95.1%		
**E465**		E	94.0%	96.9%		
**R466**		R	95.2%	97.5%		
I468		I	55.4%	77.6%		
**S469**		S	92.8%	96.3%		
**T470**		T	16.8%	58.3%		
**E471**		E	22.3%	61.1%		
**E516**		E	95.2%	97.5%		
**L518**		L	95.2%	97.5%		
H519		H	0%	49.9%		
**A520**	99.7%	A	89.2%	94.5%		
N	2,216,094		83			

* Conservation of 7D6 and 6D6 epitope in *Sarbecovirus* include clade 1, 2, 3. The epitopes of 7D6 and 6D6 in SARS-CoV-2 (N=2,216,094) were calculated according to GISAID on 11 July 2021 (https://www.gisaid.org/hcov19-mutation-dashboard/).

[#] Number in bold denoted the 7D6/6D6 sites.

Table S5 Cross-reactive or cross-neutralizing antibodies against SARS-CoV-2 and SARS-CoV.

Antibodies	Source	Reactivity	PDB no. for immune complex	Resolution (Å)	Antibody footprint on SARS-CoV-2 RBD	Identity	Reference
EY6A	Human	Cross-reactive	6ZCZ	2.64	L369, Y370, N371, F375, S376, T377, F378, K379, C380, Y381, G382, V383, S384, P385, T386, K387, N389, D390, L391, F393, A411, P412, G413, Q414, D427, D428, F429, T430, F515, F517,	28/30 (93%)	(*1*)
S304	Human	Cross-reactive	7JW0	4.30	Y369, N370, F377, K378, C379, Y380, G381, V382, S383, P384, T385, K386, N388, L390, F392, P412, G413, Q414, P426, D427, D428, F429, T430, F515, L517	23/25 (92%)	(*2*)
CR3022	Human	Cross-neutralizing	6W41	3.10	Y369, N370, S371, A372, P374, S375, T376, P377, K378, C379, Y380, G381, V382, S383, P384, T385, K386, D389, L390, P392, D427, D428, P429, T430, P515, E516, L517, H519	24/28(86%)	(*3*)
H014	Humanized	Cross-neutralizing	7CAI	3.49	Y369, A372, S373, F374, S375, T376, F377, K378, C379, Y380, S383, T385, K386, D405, V407, R408, A411, P412, G413, N439, V503	17/21(81%)	(*4*)
S309	Human	Cross-neutralizing	6WPT	3.70	T333, N334, L335, P337, G339, E340, V341, N343, A344, T345, R346, E354, K356, R357, I358, S359, N360, C361, N440, L441, K443	17/22 (77%)	(*5*)
VHH-72	Camelid	Cross-neutralizing	6WAQ	2.20	L368, Y369, N370, S371, A372, S373, F374, S375, T376, F377, K378, C379, V382, P384, T385, G404, D405, V407, R408, W436, N437, N439, V503, G504, Y508	22/27 (81%)	(*6*)
COVA1-16	Human	Cross-neutralizing	7JMW	2.89	L368, Y369, S371， F374, S375， T376， F377， K378， C379， Y380, G381， V382, S383, P384, T385, R408， Q409, P412， G413， Q414, T415, G416, D427, D428， F429	18/25（72%）	(*7*)
7D6	Mouse	Cross-neutralizing	7EAM	1.40	R346, Y351, A352, W353, N354, R355, R357, T393, N394, Y396, K462, P464, E465, R466, S469, T470, E471, E516, L518, H519, A520	16/21(76%)	This study
6D6	Mouse	Cross-neutralizing	7EAN	1.92	Y351, A352, W353, N354, R355, K356, R357, N394, Y396, P426, R457, S459, N460, K462, P463, P464, E465, R466, I468, S469, T470, E471, E516, L518, A520	20/25(80%)	This study

Table S6 Amino acid sequence of 7D6 and 6D6 antibodies.

Antibodies		Sequence
7D6	Variable Region of Light Chain	DIQMTQSPASLSASVGETVTITCRASGNIHNYLAWYQQKQGKSPQLLVYNAKTLADGVPSRFSGSGSGTQYSLKINSLQPEDFGSYYCQHFWSTPPWTFGGGTKLEVK
	Variable Region of Heavy Chain	EVQLQQSGAELVRPGASVKLSCTASGFNIKDTYIHWVKQRPEQGLEWIGRIDPGDGDTEYDPSFQGKATITADTSSNTAYLELSSLTSEDTAVYYCTRFYDYVDYGMDYWGQGTSVTVSS
6D6	Variable Region of Light Chain	DIVMTQSQKFMSTSVGDRVSVTCKASQNVGTHVAWYQQKPGQSPKALIYSASYRYSGVPDRFTGSGVGTDFTLTITNVQSEDLAEYFCQQYNSYFTFGSGTKLEIK
	Variable Region of Heavy Chain	EVQLQQSGAELVKPGASVKLSCTTSGFNIIDTYMHWVKQRPEEGLEWIGGIDPVNGNSEYDPKFQDKATITADTSSNTAYLHLSRLTSEDTAVYYCASAHYYGSSSSFPYWGQGTLVTVSA

References

1. D. Zhou *et al.*, Structural basis for the neutralization of SARS-CoV-2 by an antibody from a convalescent patient. *Nat Struct Mol Biol* **27**, 950-958 (2020).doi:https://doi.org/10.1038/s41594-020-0480-y
2. L. Piccoli *et al.*, Mapping Neutralizing and Immunodominant Sites on the SARS-CoV-2 Spike Receptor-Binding Domain by Structure-Guided High-Resolution Serology. *Cell* **183**, 1024-1042.e1021 (2020).doi:https://doi.org/10.1016/j.cell.2020.09.037
3. M. Yuan *et al.*, A highly conserved cryptic epitope in the receptor binding domains of SARS-CoV-2 and SARS-CoV. *Science* **368**, 630-633 (2020).doi:https://doi.org/10.1126/science.abb7269
4. Z. Lv *et al.*, Structural basis for neutralization of SARS-CoV-2 and SARS-CoV by a potent therapeutic antibody. *Science* **369**, 1505-1509 (2020).doi:https://doi.org/10.1126/science.abc5881
5. D. Pinto *et al.*, Cross-neutralization of SARS-CoV-2 by a human monoclonal SARS-CoV antibody. *Nature* **583**, 290-295 (2020).doi:https://doi.org//10.1038/s41586-020-2349-y
6. D. Wrapp *et al.*, Structural Basis for Potent Neutralization of Betacoronaviruses by Single-Domain Camelid Antibodies. *Cell* **181**, 1436-1441 (2020).doi:https://doi.org/10.1016/j.cell.2020.05.047
7. H. Liu *et al.*, Cross-Neutralization of a SARS-CoV-2 Antibody to a Functionally Conserved Site Is Mediated by Avidity. *Immunity* **53**, 1272-1280.e1275 (2020).doi:https://doi.org//10.1016/j.immuni.2020.10.023
