# Supplementary material for: Cross-neutralizing antibodies bind a SARS-CoV-2 cryptic site and resist circulating variants
Source: Nat Commun. 2021 Sep 27;12:5652. doi: 10.1038/s41467-021-25997-3 (PMC8476643; doi:10.1038/s41467-021-25997-3)
Supplement: Supplementary file 3 — Reporting Summary [file 41467_2021_25997_MOESM3_ESM.pdf]

nature portfolio

Corresponding author(s): Z.Hong Zhou, Zheng Zhang, Shaowei Li, Ying Gu, Ningshao Xia

Last updated by author(s): Aug 24, 2021

# Reporting Summary

Nature Portfolio wishes to improve the reproducibility of the work that we publish. This form provides structure for consistency and transparency in reporting. For further information on Nature Portfolio policies, see our Editorial Policies and the Editorial Policy Checklist.

## Statistics

For all statistical analyses, confirm that the following items are present in the figure legend, table legend, main text, or Methods section.

| n/a | Confirmed | |
|---|---|---|
| ☐ | ☒ | The exact sample size (*n*) for each experimental group/condition, given as a discrete number and unit of measurement |
| ☐ | ☒ | A statement on whether measurements were taken from distinct samples or whether the same sample was measured repeatedly |
| ☒ | ☐ | The statistical test(s) used AND whether they are one- or two-sided<br>*Only common tests should be described solely by name; describe more complex techniques in the Methods section.* |
| ☒ | ☐ | A description of all covariates tested |
| ☒ | ☐ | A description of any assumptions or corrections, such as tests of normality and adjustment for multiple comparisons |
| ☐ | ☒ | A full description of the statistical parameters including central tendency (e.g. means) or other basic estimates (e.g. regression coefficient) AND variation (e.g. standard deviation) or associated estimates of uncertainty (e.g. confidence intervals) |
| ☒ | ☐ | For null hypothesis testing, the test statistic (e.g. *F*, *t*, *r*) with confidence intervals, effect sizes, degrees of freedom and *P* value noted<br>*Give P values as exact values whenever suitable.* |
| ☒ | ☐ | For Bayesian analysis, information on the choice of priors and Markov chain Monte Carlo settings |
| ☒ | ☐ | For hierarchical and complex designs, identification of the appropriate level for tests and full reporting of outcomes |
| ☒ | ☐ | Estimates of effect sizes (e.g. Cohen's *d*, Pearson's *r*), indicating how they were calculated |

*Our web collection on statistics for biologists contains articles on many of the points above.*

## Software and code

Policy information about availability of computer code

| | |
|---|---|
| Data collection | SPR assay: Biacore 8K control solfware (2.0.15.12933);<br>HPLC: Waters Empower 3; BD FACSDiva Software v9.0;<br>X-ray diffraction data were collected at Shanghai Synchrotron Radiation Facility (SSRF) beamline BL17U1 using XDS Program Package (Jan 31, 2020); Cryo-EM data were collected using the commercial software EPU (Version 1.11); |
| Data analysis | Biacore Insight Evaluation 1.0.5.11069; GraphPad Prism 8.3.1; Phenix version 1.10.1-2155 includes Phaser and Molprobity package; COOT 0.8.9 ; MotionCor2; Gctf v1.18; CryoSPARC v2 ; Pymol version 2.1; Columbus Software 2.5.0; FlowJo version 10; |

For manuscripts utilizing custom algorithms or software that are central to the research but not yet described in published literature, software must be made available to editors and reviewers. We strongly encourage code deposition in a community repository (e.g. GitHub). See the Nature Portfolio guidelines for submitting code & software for further information.

## Data

Policy information about availability of data

All manuscripts must include a data availability statement. This statement should provide the following information, where applicable:

- Accession codes, unique identifiers, or web links for publicly available datasets
- A description of any restrictions on data availability
- For clinical datasets or third party data, please ensure that the statement adheres to our policy

The coordinates and structure factors for 7D6:RBD, 6D6:RBD have been deposited in the Protein Data Bank (accession nos. 7EAM, 7EAN).

# Field-specific reporting

Please select the one below that is the best fit for your research. If you are not sure, read the appropriate sections before making your selection.

- [x] Life sciences
- [ ] Behavioural & social sciences
- [ ] Ecological, evolutionary & environmental sciences

For a reference copy of the document with all sections, see nature.com/documents/nr-reporting-summary-flat.pdf

# Life sciences study design

All studies must disclose on these points even when the disclosure is negative.

| | |
|---|---|
| Sample size | Sample sizes were estimated based on experiences published in the literature (DOI: 10.1126/science.aao7298, 10.1126/science.aao7283). |
| Data exclusions | No data were excluded from the analyses. |
| Replication | Experimental findings were reliably reproduced. Most of the experiments were replicated two times. |
| Randomization | Immune complex particles were sparsely and randomly distributed in the CryoEM grids during data collection. |
| Blinding | Data collection and analysis were performed by different people, the sample classification were placed by simple marks during analysis. |

# Reporting for specific materials, systems and methods

We require information from authors about some types of materials, experimental systems and methods used in many studies. Here, indicate whether each material, system or method listed is relevant to your study. If you are not sure if a list item applies to your research, read the appropriate section before selecting a response.

## Materials & experimental systems

| n/a | Involved in the study | |
|---|---|---|
| [ ] | [x] | Antibodies |
| [ ] | [x] | Eukaryotic cell lines |
| [x] | [ ] | Palaeontology and archaeology |
| [ ] | [x] | Animals and other organisms |
| [x] | [ ] | Human research participants |
| [x] | [ ] | Clinical data |
| [x] | [ ] | Dual use research of concern |

## Methods

| n/a | Involved in the study | |
|---|---|---|
| [x] | [ ] | ChIP-seq |
| [ ] | [x] | Flow cytometry |
| [x] | [ ] | MRI-based neuroimaging |

## Antibodies

| | |
|---|---|
| Antibodies used | Primary antibodies:<br>(1) Antibodies against SARS-CoV-2 proteins were prepared in mice from our own lab, including: 7D6, 5A12, 13F1, 12F7, 12F9, 6D6, 16D8, 19F10, 17F10, 4A5, 10D1, 20H2, 8G5, 5E7, 1B11;<br>(2) Antibody CR0322 and REGN10933 were expressed fin CHO.<br>(3) Peroxidase (HRP)-labeled goat anti-mouse antibody (Abcam, Cat#ab97240, 1:5000 dilution)), goat anti-human antibody (Abcam, Cat#ab97225,1:5000 dilution), rabbit anti-SARS-CoV-2 NP IgG (Sino Biological, Cat#40143-R004), HRP-conjugated goat anti-rabbit IgG (H+L) antibody (TransGen Biotech, Cat#HS101-01, 1:5000 dilution), goat anti-mouse IgG (H + L) Alexa Flour 647 (Thermo Fisher, A-21236, 1:5000 dilution) |
| Validation | The activities of the monoclonal antibodies (Mouse antibodies: 7D6, 5A12, 13F1, 12F7, 12F9, 6D6, 16D8, 19F10, 17F10, 4A5, 10D1, 20H2, 8G5, 5E7, 1B11; Human antibodies : CR0322 and REGN10933 ) were validated by ELISA, pseudotype LV, VSV-based neutralization assay or authentic SARS-CoV-2 neutralization assay, which are provided in the manuscript.<br>We followed the manufactures' instruction to use the below listed antibodies. All antibodies work well.<br>1. Peroxidase (HRP)-labeled goat anti-mouse antibody (Goat antibody, specific for mouse; https://www.abcam.com/goat-mouse-igg1-hrp-ab97240.html);<br>2. Goat anti-human antibody (Goat antibody, specific for human; https://www.abcam.com/goat-human-igg-fc-hrp-ab97225.html);<br>3. Rabbit anti-SARS-CoV-2 NP IgG (Rabbit antibody; https://cn.sinobiological.com/antibodies/cov-nucleocapsid-40143-r004);<br>4. HRP-conjugated goat anti-rabbit IgG (H+L) antibody (Goat antibody, specific for rabbit; https://www.transgen.com.cn/antibody_second/397.html);<br>5. Goat anti-mouse IgG (H + L) Alexa Flour 647 (Goat antibody, specific for mouse; https://www.thermofisher.cn/cn/zh/antibody/product/Goat-anti-Mouse-IgG-H-L-Highly-Cross-Adsorbed-Secondary-Antibody-Polyclonal/A-21236); |

# Eukaryotic cell lines

Policy information about cell lines

| | |
|---|---|
| Cell line source(s) | H1299-ACE2hR cells were constructed in our laboratory. The H1299-ACE2hR which stably over-expressed human ACE2 and and nuclear-localizedRFP was constructed by lentiviral transduction based on H1299 cells. The HEK293T, H1299, BHK21 and Vero E6 were purchased from ATCC. The CHO, sf9 and Hive Five cells were purchased from Thermo Fisher Scientific. |
| Authentication | The cell lines were not authenticated since they were purchased commercially. |
| Mycoplasma contamination | The cell lines were tested to be negative for mycoplasma contamination prior to experiments. |
| Commonly misidentified lines (See ICLAC register) | No commonly misidentified cell lines were used in this study. |

# Animals and other organisms

Policy information about studies involving animals; ARRIVE guidelines recommended for reporting animal research

| | |
|---|---|
| Laboratory animals | 6-8-week-old, female BALB/c mice were used in this study. |
| Wild animals | The study did not involve wild animals. |
| Field-collected samples | The study did not involve samples collected from the field. |
| Ethics oversight | All procedures in this study involving the authentic COVID-19 virus were performed in a biosafety level 3 (BSL-3) facility of the Shenzhen Third People's Hospital, China. The experimental protocols were approved by the Xiamen University Laboratory Animal Management Ethics Committee. All manipulations were strictly conducted in compliance with animal ethics guidelines and approved protocols. |

Note that full information on the approval of the study protocol must also be provided in the manuscript.

# Flow Cytometry

## Plots

Confirm that:

- [x] The axis labels state the marker and fluorochrome used (e.g. CD4-FITC).
- [x] The axis scales are clearly visible. Include numbers along axes only for bottom left plot of group (a 'group' is an analysis of identical markers).
- [x] All plots are contour plots with outliers or pseudocolor plots.
- [x] A numerical value for number of cells or percentage (with statistics) is provided.

## Methodology

| | |
|---|---|
| Sample preparation | Cell sample were prepared as described in the methodology.In brief, plasmids encoding SARS-CoV-2 wildtype S were transfected into HEK293T cells. Cells samples were prepared in multiples for serial incubation with IgG or Fabs (1 mg/mL) at 37 °C for 120, 60 and 5 min. Immediately after the incubation time, cells were transferred to ice then thoroughly washed with ice-cold PBS and 2% FBS. Samples were then stained with anti-mouse IgG (H + L) Alexa Flour 647 (Thermo Fisher) for 30 min. After thorough washes with ice-cold PBS and 2% FBS, samples were resuspended and analyzed with FACS Calibur (BD Biosciences, USA) and FlowJo 10 software (FlowJo, USA). |
| Instrument | FACS Calibur (BD Biosciences) |
| Software | FlowJo version 10 |
| Cell population abundance | No sorting was performed with the flow cytometer. |
| Gating strategy | Cells were first gate by FSC and SSC to obtain single cells, and then gated for Alexa Flour 647 Positive versus Negative. |

- [ ] Tick this box to confirm that a figure exemplifying the gating strategy is provided in the Supplementary Information.
